# An experimental investigation into the rheological behavior and filtration loss properties of water-based drilling fluid enhanced with a polyethyleneimine-grafted graphene oxide nanocomposite

**DOI:** 10.1039/d3ra07874d

**Published:** 2024-04-03

**Authors:** Abdul Hazim Abdullah, Syahrir Ridha, Dzeti Farhah Mohshim, Mohd Azuwan Maoinser

**Affiliations:** a Department of Petroleum Engineering, Universiti Teknologi PETRONAS Seri Iskandar Perak 32610 Malaysia syahrir.ridha@utp.edu.my; b Institute of Hydrocarbon Recovery, Universiti Teknologi PETRONAS Seri Iskandar Perak 32610 Malaysia

## Abstract

The modern oil and gas industry, driven by a surging global energy demand, faces the challenge of exploring deeper geological formations. Ensuring the robust performance of drilling fluids under harsh wellbore conditions is paramount, with elevated temperatures and salt contamination recognized as detrimental factors affecting the rheological and filtration loss properties of drilling fluids. We successfully synthesized a polyethyleneimine-grafted graphene oxide nanocomposite (PEI-GO), and its functional groups formation and thermal stability were verified through Fourier Transform Infrared Spectroscopy (FTIR) and Thermogravimetric Analysis (TGA). Our findings demonstrated a significant improvement in the plastic viscosity and yield point of the base drilling fluid with the addition of PEI-GO. The inclusion of 0.3 wt% PEI-GO outperformed the base drilling fluid at 160 °C, improving the yield point/plastic viscosity (YP/PV) value and reducing filtration loss volume by 42% and 67%, respectively. The Herschel–Bulkley model emerged as the superior choice for characterizing rheological behavior. PEI-GO exhibited compatibility with high-salt formations, maintaining satisfactory filtration volumes even when subjected to sodium chloride (NaCl) and calcium chloride (CaCl_2_) contamination concentrations of up to 20 and 10 wt%, respectively. The remarkable rheological and filtration properties of PEI-GO are attributed to its electrostatic interactions with clay particles through hydrogen and ionic bonding. These interactions lead to pore plugging in the filter cake, effectively preventing water infiltration and reducing filtration loss volume. This study emphasizes the potential of PEI-GO in water-based drilling fluids, particularly in high-temperature and salt-contaminated environments.

## Abstract 2

The assessment of the impact of polyethyleneimine-grafted graphene oxide (PEI-GO) nanocomposite on rheological and filtration loss properties offers fresh perspectives on its potential as an additive in water-based drilling fluids applications.

## Graphical abstract

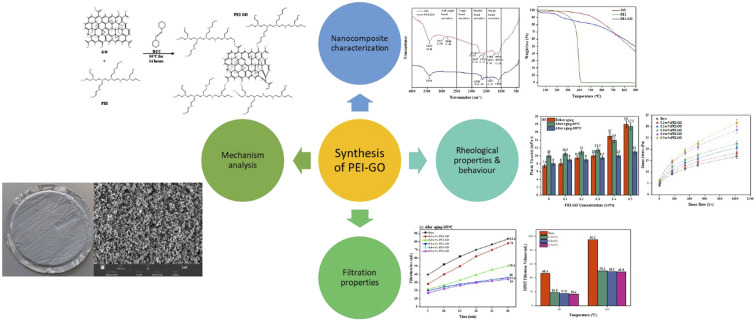

## 1. Introduction

Drilling fluids are a key aspect in the success of oil and gas exploration and drilling operations. These fluids can be categorized into water-based, oil-based, and synthetic-based fluids, with water-based fluids being preferred to reduce high operational cost and because of their cost-effectiveness and environmental safety. However, water-based fluids impose certain wellbore issues owing to water's reactivity with reactive formations like shale. The water might intrude into the formation, leading to shale hydration and swelling, weakened formations, and wellbore collapse.^1,2^ Simultaneously, the water loss can result in the formation of a dense filter cake on the borehole wall, causing borehole damage and pipe sticking.^3^ To avoid these issues, it is crucial to control drilling fluids' properties, particularly rheology and filtration loss, to ensure efficient drilling operations.^4^ Rheological properties determine the fluid's flow behavior and its ability to suspend and transport cuttings, while filtration loss properties dictate the fluid's ability to control fluid loss into the formation.^5,6^ Stable rheological features and low fluid loss in downhole conditions were crucial for maintaining well stability, preventing formation damage, and optimizing drilling productivity. Consequently, rheology modifiers and fluid loss additives such as surfactants, polymers, and nanoparticles were incorporated into the formulation to improve the rheological properties and minimize the fluid loss to the formation.^7,8^

Polyethyleneimine (PEI) is categorized as a cationic polymer, indicating its positivity is derived from the inclusion of amino groups (–NH_2_) within its polymer chain, rendering it cationic in nature. This property, stemming from the amino groups, further underlined the feasibility of employing positively charged polymers as drilling fluid's additive within high-temperature reservoirs. The interaction of electrostatic forces among particles within drilling fluid is recognized as a major factor in improving rheological properties.^9^ Branched PEI exhibited greater efficacy in inhibiting clay hydration and swelling when compared to 1,6-hexamethylenediamine.^10^ The addition of less than 1% branched PEI resulted in enhancements in rheological parameters and effective control of fluid loss. In a different study, PEI with high molecular weight had better shale inhibition capability but had worse water solubility and increased cost.^11,12^ Although PEI had the capability to engage with clay *via* hydrogen bonding and electrostatic interactions, it exhibits limited resilience when exposed to temperatures exceeding 120 °C.^12^ Meanwhile, only a slight improvement in viscosity was observed when PEI was used to formulate a polymer-based drilling fluid.^13,14^ To overcome these limitations, nanoparticles such as silica and graphene oxide (GO) were grafted with PEI to improve their properties.^15–17^

In recent years, the application of polymer nanocomposite (PNC) in drilling fluid has been investigated to improve rheological properties, filter loss control and shale inhibition.^18,19^ PNC encompasses inorganic nanoparticles that are either grafted with polymer chains on their surface or are integrated within a polymer matrix.^19,20^ This amalgamation capitalizes on the attributes of both elements, thereby heightening the material properties of the resultant product. These PNCs were formed through various polymerization methods such as radical, adsorption of polymeric dispersants, and *in situ* surface modification.^21^ Furthermore, comprehending the phase behavior of polymer nanocomposites holds paramount importance in evaluating their thermal degradation tendencies. Siddique *et al.*^22^ investigated the structural and thermal degradation patterns of reclaimed clay nano-reinforced low-density polyethylene nanocomposites. Their study underscored the significance of discerning distinct phases within the polymer matrix, particularly the crystalline and amorphous phases. These structural attributes of the polymer, such as the degree of substitution and molar mass, exhibit multifunctional behavior, exerting influence over various properties of the drilling fluid.^23^ The phase behavior of polymer nanocomposites within drilling fluids is pivotal for optimizing drilling fluid performance. A profound understanding of the diverse phases inherent in the polymer matrix, coupled with judicious nanoparticle selection and achieving uniform dispersion, can yield significant enhancements in the rheological, thermal, and filtration properties of the drilling fluid.

The polyethyleneimine-graphene oxide (PEI-GO) composite belongs to the category of PNCs and has been investigated for its potential as a rheology enhancer and fluid loss reducer in drilling fluids. The findings from ref. 16 demonstrated a slight improvement in the rheological properties of PEI-GO in comparison to the base drilling fluid. However, this study is limited to a single concentration and a lower aging temperature of 65 °C. A recent study by Zhao *et al.*^17^ explored different PEI-GO and montmorillonite concentrations, along with elevated aging temperatures up to 150 °C, regarding rheology and American Petroleum Institute (API) filtration loss. The results indicated that the montmorillonite solution with PEI-GO had lower plastic viscosity (PV) and exhibited the lowest yield point (YP), compared to most inhibitors except potassium chloride (KCl) which signified improved rheological behavior. The inhibitor's compatibility with drilling fluids was evident in the viscosity and API filtration loss differences before and after its addition. Notably, PEI-GO's influence on drilling fluid's reliability was minimal post-aging, suggesting good compatibility with standard drilling fluid additives.

**Fig. 1:**
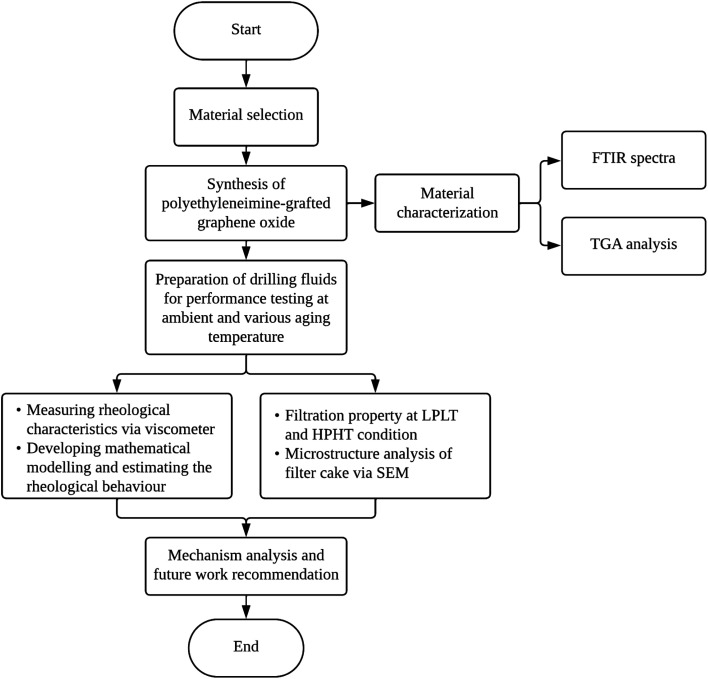
Flowchart outlining the experimental procedures conducted in this study.

The utilization of the PEI-GO in drilling fluids has been a subject of considerable interest and research in recent years. The motivation of this study lies in addressing the following critical gaps in the current understanding of PEI-GO as a rheology modifier and filtration loss control agent in water-based drilling fluids. Despite the available literature, there has been a limited study to determine the most suitable rheological model for describing the flow behavior of this novel PNC. In addition, the suitability of PEI-GO under high salt concentration is yet to be studied. Examining the drilling fluid's tolerance in high salinity is crucial, as it has the potential to degrade the fluids' rheological characteristics and diminish their stability to high temperatures. Further research is needed to investigate the PEI-GO's specific influence on rheological behavior and filtration loss performance, particularly concerning HPHT conditions and salt contamination. This study aims to examine the performance of PEI-GO in water-based drilling fluids under elevated aging temperatures and high salinity conditions, with a focus on its rheological and filtration properties. Additionally, we also assess the suitability of various rheological models in describing the flow behavior of this modified drilling fluid. To ensure the effective organization of the discussion, this article is divided into subsections. The flowchart in Fig. 1 illustrates the structure and flow of the research, encompassing the experimental design, data collection, and analysis.

## 2. Materials and methods

### 2.1 Materials

Functionalized GO, PEI and dicyclohexylcarbodiimide (DCC) were procured for the synthesis of PEI-GO. The composition of water-based drilling fluids comprised of varied chemical additives: bentonite, KCl, xanthan gum (XG), carboxymethyl cellulose (CMC), sodium hydroxide (NaOH), and barite. These additives were formulated in a specific sequence and duration to achieve the desired formulation. Sodium chloride (NaCl) and calcium chloride (CaCl_2_) were acquired to examine the impact of salt contamination on drilling fluid performance. NaCl and CaCl_2_ were chosen as materials in salt contamination analysis of drilling fluid due to their ability to mimic the salt content in oil basins, their impact on rheological behavior and their interaction with polymers and biopolymers in drilling fluids.^24,25^ All materials were commercially available and procured from Sigma-Aldrich.

### 2.2 Methods

#### 2.2.1 Synthesis of PEI-GO

**Fig. 2:**
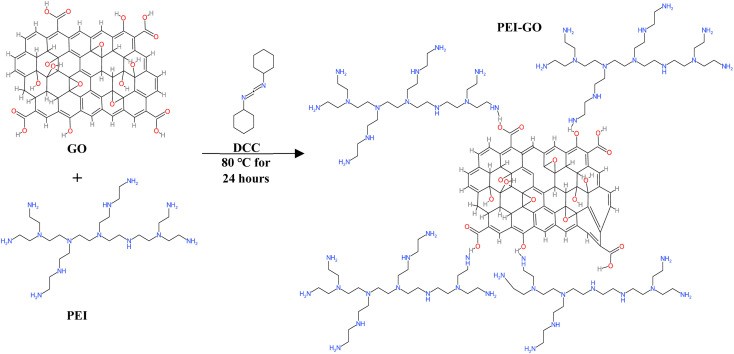
Synthesis process of PEI-GO.

PEI-GO composite was synthesized *via* the *in situ* solution polymerization method.^16^ The GO nanosheets were first dispersed in deionized water with a concentration of 1 mg mL^−1^ by ultrasonication at 40 kHz and 350 W for 30 minutes. The resultant GO nanofluid and a controlled amount of PEI solution were mixed in a beaker using a magnetic stirrer hot plate. Next, DCC was slowly added to the solution to act as a cross-linking reagent for the PEI-GO polymerization process. The system was kept under continuous stirring at a constant temperature of 60 °C for 24 hours. Then, the solution was centrifuged at 15 000 rpm for 30 minutes with ethanol to separate the PEI-GO from the residual polymer. Finally, the PEI-GO was subjected to drying in an oven set at 80 °C for 4 hours and the PEI-GO powder was collected and used for characterization analysis and performance evaluation. The schematic representation of the PEI-GO synthesis process is depicted in Fig. 2.

#### 2.2.2 Preparation of water-based drilling fluids

The base drilling fluid was formulated by mixing distilled water with bentonite, KCl, XG, CMC, NaOH, and barium sulfate, following specific mixing times and order, as well as their designated functions outlined in Table 1. While the addition of additives to drilling fluids lacks an exact standard, their introduction aimed to maintain the total fluid volume under 350 mL, in accordance with API standard.^26^ The addition of NaOH was implemented to increase the pH, aligning with the common pH range of 8–11 in drilling fluids. This adjustment enhances bentonite dispersion, facilitates sufficient dissolution of additives in the drilling fluids, and minimizes corrosiveness to drilling tools as recommended in literature.^27^

**Table 1:** Formulation of drilling fluids

Materials	Quantity	Mixing time (min)	Mixing order	Functions
Distilled water (mL)	315.0	—	1	Base fluid
Bentonite (g)	22.5	5	2	Viscosifier
KCl (g)	20.0	3	3	Shale inhibitor
XG (g)	1.0	3	4	Rheology modifier
CMC (g)	1.5	2	5	Filtration loss agent
NaOH (g)	0.5	2	6	Control pH
Barium sulfate (g)	60.0	30	7	Weighting agent
PEI-GO (wt%)	0.1–0.5	10	8	Rheology modifier and filtration loss agent

Before blending the nanocomposite with the base drilling fluid, the PEI-GO sample underwent a preparation process using the ultrasonication technique to prevent aggregation.^28,29^ Initially, the PEI-GO was dispersed in deionized water using an ultrasonic homogenizer for 30 minutes. Subsequently, it was introduced into the mixing cup to generate drilling fluids with varying PEI-GO concentrations. The solution underwent an additional 10 minutes of mixing to achieve a homogeneous drilling fluid mixture. All formulations aimed to yield 350 mL of water-based drilling fluids with a density of 9.5 ppg. Achieving a consistent 9.5 ppg density across different formulations might necessitate minor adjustments in the quantities of distilled water and barium sulfate, owing to variations in PEI-GO concentrations.^30^ These drilling fluid samples were placed in an aging cell under 500 psi and subjected to temperature aging at 80 and 160 °C for 16 hours to replicate the borehole's fluid circulation and to investigate the effect of temperature and salt resistance on drilling fluid's rheology and filtration loss properties.

### 2.3 Material characterization

#### 2.3.1 Fourier transform infrared spectroscopy (FTIR)

FTIR analysis was conducted to confirm the successful grafting of PEI molecules onto the GO surface and study the chemical structure of PEI-GO. The analysis was accomplished using PerkinElmer Frontier-01 FTIR equipment, which had a wavelength range of 4000 to 400 cm^−1^. Data were collected at a spectral resolution of 1 cm^−1^ under ambient conditions. The FTIR spectra obtained for GO, PEI, and PEI-GO were analyzed for characteristic peaks and bands that indicated the presence of functional groups.^31^

#### 2.3.2 Thermogravimetric analysis (TGA)

TGA was performed to assess the thermal stability of the developed PEI-GO, and a comparison between GO and PEI was established. This analysis was conducted using a PerkinElmer Simultaneous Thermal Analyzer 6000 under a nitrogen environment. Samples of GO, PEI, and PEI-GO were prepared and heated from 29 to 900 °C at a heating rate of 10 °C min^−1^, and the mass change of the samples was measured. The TGA curves were analyzed for significant differences in mass loss and thermal stability between the samples.

### 2.4 Performance evaluation

#### 2.4.1 Rheological properties

The rheological characteristics of the drilling fluids were assessed, encompassing measurements of PV and YP. These measurements were conducted utilizing a FANN rotational viscometer at six speeds, specifically 600 rpm, 300 rpm, 200 rpm, 100 rpm, 6 rpm, and 3 rpm. It is noteworthy that both pre- and post-aging assessments were performed on all drilling fluid samples. Based on API 13B-1 recommendations, the PV and YP values were subsequently calculated by employing the following formulas:^32^

1 PV = *Φ*_600_ − *Φ*_300_ (mPa s)

2 YP = 0.5 × (*Φ*_300_ − PV) (Pa)

where *Φ*_600_ and *Φ*_300_ are the viscometer dial readings at 600 rpm and 300 rpm, respectively.

#### 2.4.2 Rheological modeling

Rheological models are essential for characterizing the behavior of drilling fluids, serving as key parameters for accurately calculating pressure drop, ensuring efficient hole cleaning, and managing drilling hydraulics.^33,34^ Choosing the right rheological model reduces computational errors and improves prediction accuracy during drilling operations. Drilling fluids often display non-Newtonian behavior due to factors like clay particles and electric charges on clay surfaces, causing viscosity to change with shear rate.^8,35^ The Bingham plastic, power law, and Herschel–Bulkley models are employed to predict the rheological behavior of drilling fluid, as outlined below:

##### 2.4.2.1 Bingham Plastic Model

This model is applied to fluids demonstrating Bingham plastic behavior, characterized by linear *τ*/*γ* behavior beyond an initial shear stress threshold. The PV represents the slope of this line, and the YP signifies the threshold stress. It can be expressed as follows:^36^

3 *τ* = *τ*_0_ + *μ*_p_*γ̇*

##### 2.4.2.2 Power Law Model

Fluids exhibiting power law behavior can be characterized by the following expression:^37^

4 *τ* = *Kγ̇*^*n*^

##### 2.4.2.3 Herschel–Bulkley Model

This model also well-known as a yield power-law model, is expressed as follows:^38^

5 *τ* = *τ*_0_ + *Kγ̇*^*n*^

where *τ* is the shear stress (Pa), *τ*_0_ is YP (Pa), *γ̇* is the shear rate (1/*s*), *μ*_p_ is the PV, *K* is the fluid consistency coefficient, and *n* is the fluid behavior index. In this study, the most appropriate rheological model is determined by measuring the required rheological parameters (PV and YP) using a FANN rotational viscometer. Subsequently, these values are integrated into the rheological models to calculate the coefficient of determination (*R*^2^) to assess the degree of alignment between the obtained outcomes and the desired values, with a value nearing 1 signifying a highly accurate prediction.

#### 2.4.3 Filtration loss

API fluid loss tests were conducted for all drilling fluid samples at a standard temperature of 25 °C and pressure of 100 psi. For each sample, 350 mL of drilling fluid was loaded into the cell body, and the volume of liquid filtrate was measured every 5 minutes for 30 minutes. A high-pressure high-temperature (HPHT) fluid loss test was conducted by loading 175 mL of each drilling fluid sample into HPHT filter press cells and conducting the test at a temperature of 160 °C and a differential pressure of 500 psi. The mechanism of filtration loss improvement was analyzed using scanning electron microscopy (SEM) on filter cake samples of drilling fluids.

## 3. Results and discussions

### 3.1 Material characterization

**Fig. 3:**
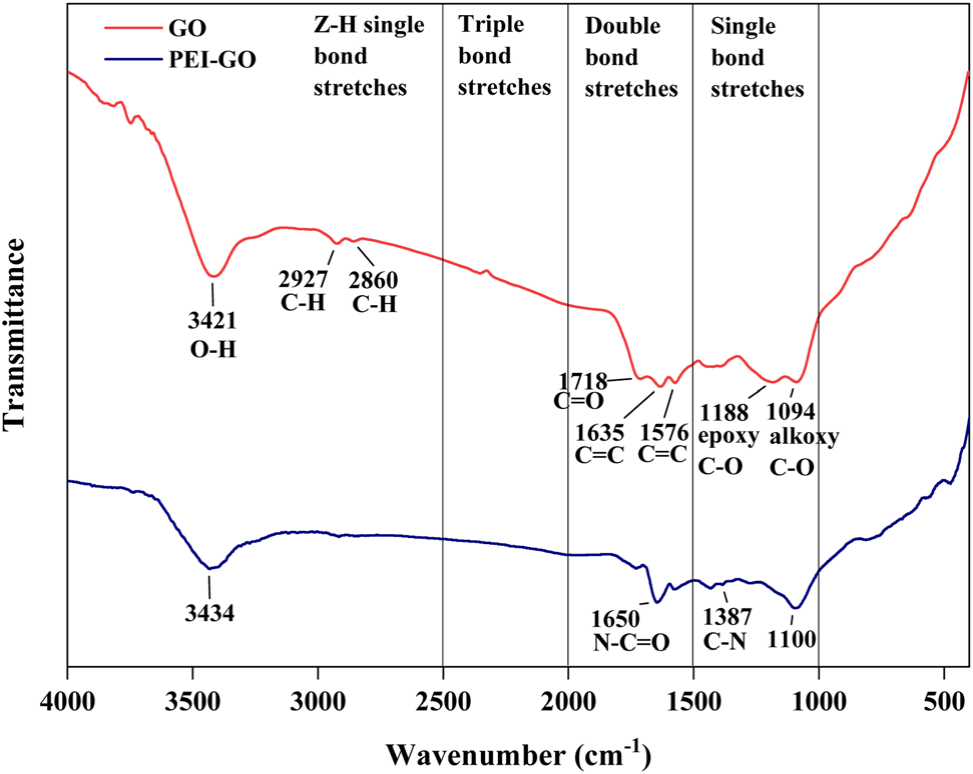
FTIR spectra of GO and PEI-GO.

Fourier Transform Infrared Spectroscopy (FTIR) analysis was performed to identify the functional group of GO and confirm the surface modification of PEI-GO as shown in Fig. 3. Based on GO peak characteristics, the broad peak located at 3421 cm^−1^ was related to the O–H stretching vibration. Two absorption spectra were observed at 2927 and 2860 cm^−1^ owing to C–H groups of graphene.^16^ The peak at 1718 cm^−1^ arose from the C=O stretching vibration in COOH groups. Two peaks located at 1635 and 1576 cm^−1^ were attributed to C=C stretching vibration. The alkoxy C–O and epoxy C–O groups were identified through the stretching peaks at 1188 and 1094 cm^−1^, respectively. All of GO's distinctive peaks found in this study were consistent with earlier findings.^39,40^ As for PEI-GO, the functionalization of PEI by grafting onto GO surfaces was demonstrated by the absence of the epoxy C–O peak at 1188 cm^−1^. The peak at wavelength 3434 cm^−1^ was attributed to the hydrogen-bonded hydroxyl group (–OH) and amino group (–NH_2_) hydrogen stretching bands.^41^ In addition. the N–C=O and C–N ionic bonds at 1650 cm^−1^ and 1387 cm^−1^ were formed, indicating the successful absorption vibration of the amide group in the molecular chain of PEI, respectively.

**Fig. 4:**
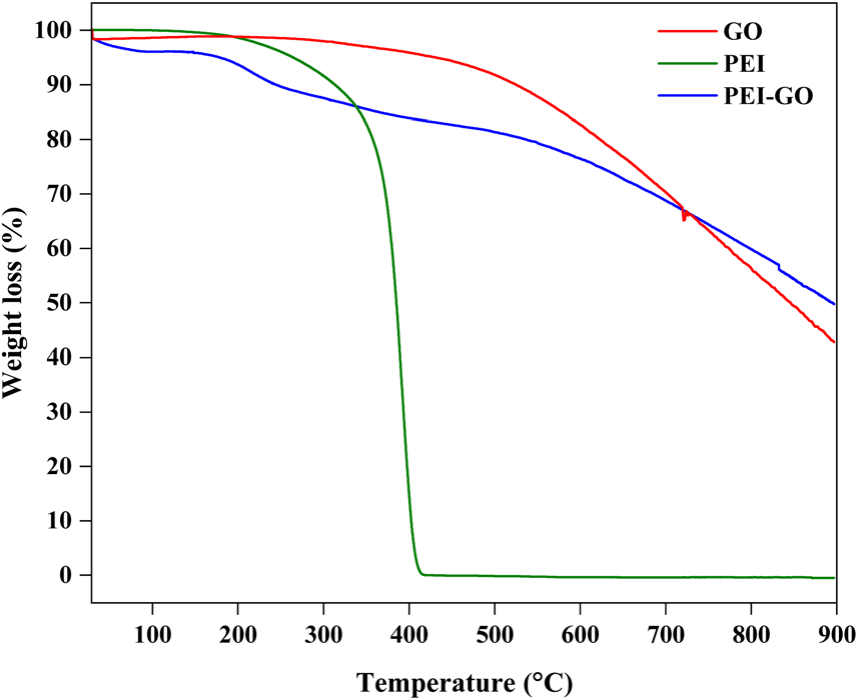
TGA curves of GO, PEI, and PEI-GO.

The TGA analysis for GO, PEI, and PEI-GO is shown in Fig. 4. PEI displayed weight loss beginning at 150 until 337 °C which accounts for 14%. Then, major weight loss was observed up to 400 °C where 86% of PEI was lost and only 0.1% residue was left until the end of the TGA test. This pattern was comparable to that seen by Liu *et al.*^42^ As for GO, no significant weight loss was observed between 30 and 285 °C. Only 6% weight loss occurs around 490 °C and the weight loss decreases linearly until 900 °C where the remaining residue for GO was around 43%. In the case of PEI-GO, a different trend of TGA was observed where weight loss was about 7% between 150 and 258 °C due to the efficient removal of oxygen-functional groups.^31,43^ At 700 °C, the intersection between GO and PEO-GO occurred and showed that the decrease in weight loss is higher for GO as compared to PEI-GO. At 900 °C, 50% of PEI-GO was lost and 50% residue was left, higher than the GO. The decrease in mass loss confirmed the thermal stability property and modification of the PEI on the GO surface.

### 3.2 Rheological properties

In this study, the rheological characteristics of drilling fluids were described through PV and YP. PV is a measure of flow resistance of drilling fluids, which is influenced by the solid content and electrochemical forces between the reactive particles.^44,45^ YP represents the initial stress required to initiate the flow of non-Newtonian fluids and indicates the drilling fluid's effectiveness in cleaning the borehole.^46^ YP is influenced by both the volume and electrochemical attractive forces among solid particles added to the drilling fluid.^47^

**Fig. 5:**
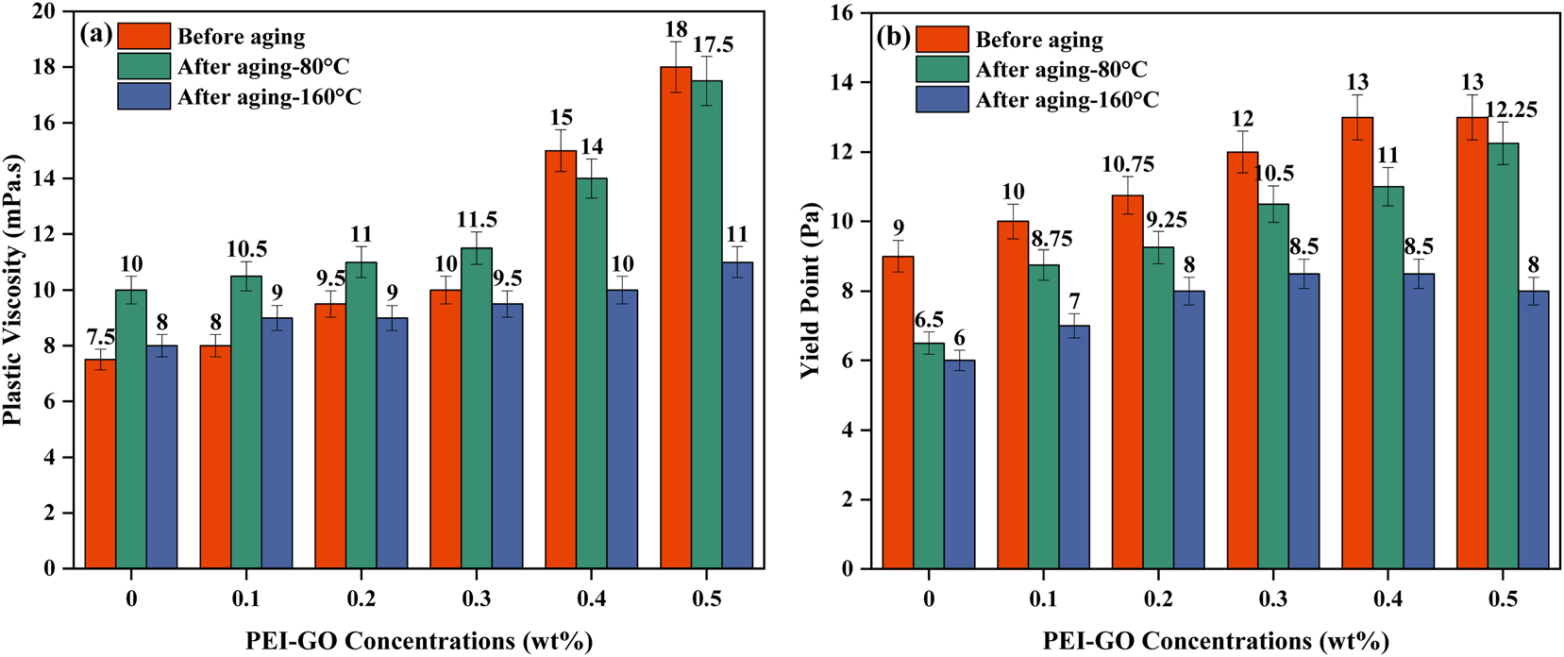
(a) Plastic viscosity and (b) yield point, of base drilling fluid with increasing PEI-GO concentration at different temperatures.

As illustrated in Fig. 5a, the PV of the drilling fluid increased with the increasing concentration of PEI-GO at ambient temperature. This finding aligns with the report made by Li *et al.*,^48^ indicating that graphene can establish strong interactions with the polymer matrix, leading to heightened viscosity. The increase in PV observed in the drilling fluids upon the addition of PEI-GO can be attributed to the formation of hydrogen bonds and amide groups' intercalation between bentonite particles and the PEI-GO nanocomposite. The presence of these functional groups has been confirmed through the earlier FTIR characterization analysis in this study. When cationic PEI-GO was introduced into the base drilling fluid, led to ion fixation and exchange which resulted in heightened electrostatic attractive forces between the negatively charged bentonite platelets and the incorporated PNCs.^49,50^ Notably, a concentration of 0.1 to 0.3 wt% PEI-GO resulted in a minimal increase in PV. However, when 0.4 wt% and 0.5 wt% PEI-GO were added, the PV experienced a significant rise to 15 and 18 mPa s. This can be attributed to stronger attraction forces between the clay surface and PNCs caused by a higher solid content, resulting in the flocculation and aggregation of PNCs. This phenomenon is caused by increased attraction, poor distribution, and reduced separation between solid components.^47^

High temperatures typically degrade polymers such as XG and CMC, leading to a decrease in rheological parameters. However, Fig. 5a shows a slight increase in the PV of the base drilling fluid after thermal aging. The addition of a low concentration of XG and CMC did not significantly affect the rheological properties of the base drilling fluid. Instead, the viscosity after aging was primarily influenced by the dispersion of bentonite in the base drilling fluid at elevated temperatures.^51^ The effect of temperature after the addition of PEI-GO can be assessed as PV increased significantly when the PEI-GO concentrations increased for drilling fluids sample before aging and after aging at 80 °C, while at 160 °C, the increase in PV was minimal. The increment of PV is happening since both side and main chains of the nanocomposite remain intact after aging. Consequently, the increased number of PEI-GO molecules, due to the extended chains enveloping the interlayer spaces of the bentonite, resulted in an increased surface area and greater flow resistance.^51^ Other studies observed that elevated temperatures led to greater kinetic activity among the solid particles present in the drilling fluid.^47^ This increased interparticle interaction resulted in the thermal dispersion of bentonite particles, ultimately contributing to the heightened viscosity of the drilling fluid sample.^49,52^ However, when comparing PV between temperatures at the same PEI-GO concentration, the PV exhibited an opposing trend. The PV of drilling fluids after aging at 80 °C is higher than that of drilling fluids before aging, especially at lower PEI-GO concentrations, with marginal differences at higher PEI-GO concentrations. In contrast, aging at 160 °C results in a reduction in PV compared to both the drilling fluids before aging and those aged at 80 °C, especially after using 0.3 to 0.5 wt% of PEI-GO. At higher temperatures, PEI-GO molecules form due to increased dissociative adsorption of water by amine groups on side chains, resulting in decreased adsorption of the polymer nanocomposite on clay particles.^51^ Nonetheless, it is advantageous to employ a drilling fluid with a lower PV value for efficient drilling operations, which can lead to an improved rate of penetration, reduced wellbore pressure, and decreased risks of formation fractures and differential pipe sticking.^53,54^ Hence, the PV remains most stable across all temperature conditions when adding up to 0.3 wt% PEI-GO, and concentrations beyond 0.4 wt% adversely affect the drilling fluid's PV.

The effect of increasing PEI-GO concentrations on the YP is exhibited in Fig. 5b. It was shown that the YP of base drilling fluids was increased with the increment of PEI-GO concentrations, as a result of PNCs addition that increased the solid content and caused the attractive electrostatic forces among the particles to amplify. This increasing trend is similar for drilling fluid samples that underwent aging at 80 and 160 °C. The finding also showed that higher temperatures caused the YP to drop at the same PEI-GO concentrations. The lower YP at higher temperatures is a result of the thermal degradation of solid particles in the drilling fluid, which increases the distance between molecules and reduces flow resistance.^55^ Having a high YP in drilling fluids is advantageous as it signifies improved cutting transport. Nonetheless, an excessively high YP can negatively impact the cutting carrying capacity, whereas an excessively low YP can lead to the sagging of barite and drill cuttings.^44^ Therefore, it is recommended to find the optimal formulation by analyzing the YP/PV ratio of the drilling fluid.

The YP/PV ratio serves as a crucial indicator of shear-thinning behavior and the overall carrying capacity of the drilling fluid.^47,56^ This ratio plays a pivotal role in optimizing drilling fluid efficiency, favoring a combination of low plastic viscosity and high yield point. A higher YP/PV value not only signifies improved shear-thinning behavior but also enhances the fluid's ability to efficiently transport rock cuttings at low shear rates and facilitate effective rock fragmentation at high shear rates.^57,58^ This balance in rheological properties is instrumental in achieving optimal drilling performance. Table 2 provides the YP/PV ratios for drilling fluids with increasing concentrations of PEI-GO at varying aging temperatures. The highest YP/PV ratios are observed at 0.1 wt% PEI-GO before aging and at 0.3 wt% PEI-GO after aging at 80 °C. Additionally, maximum YP/PV values are found at 0.2 wt% and 0.3 wt% PEI-GO after aging at 160 °C. It can be concluded that the concentration of 0.3 wt% PEI-GO is the most effective among various concentrations, exhibiting a consistently high YP/PV value, even under elevated temperatures of 80 and 160 °C. The exceptional performance of PEI-GO under elevated temperatures suggests a high degree of thermal stability. In summary, the comparison of rheological properties demonstrates a significant enhancement and stability in the rheology of drilling fluids with the addition of PEI-GO, irrespective of temperature conditions.

**Table 2:** YP/PV of the drilling fluid with increasing PEI-GO concentrations at different temperature conditions

PEI-GO concentrations (wt%)	Before aging	After aging-80 °C	After aging-160 °C
0	1.20	0.65	0.75
0.1	1.25	0.83	0.78
0.2	1.13	0.84	0.89
0.3	1.20	0.91	0.89
0.4	0.87	0.79	0.85
0.5	0.72	0.70	0.73

### 3.3 Rheological modeling and shear thinning behaviour

**Fig. 6:**
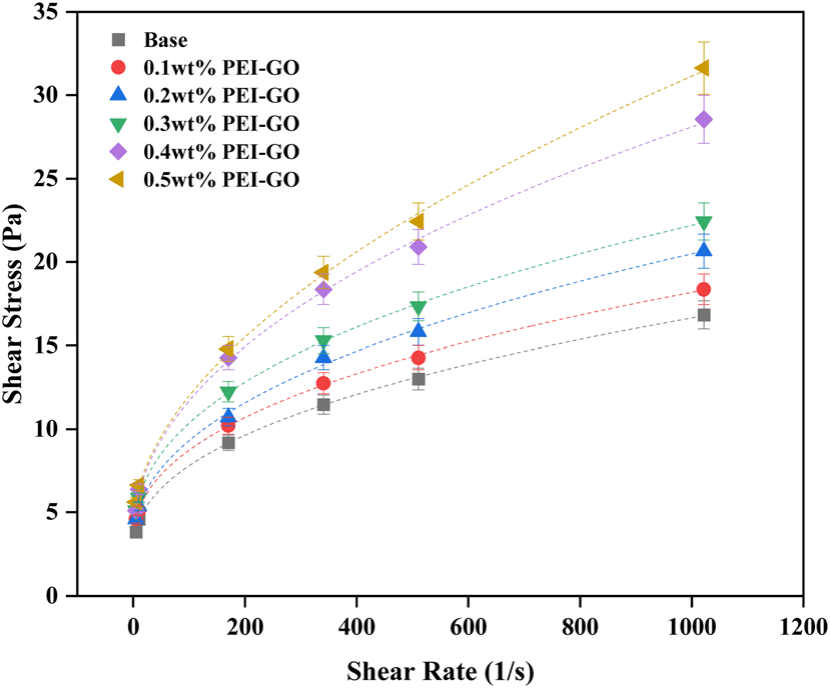
Rheogram of base drilling fluids with various PEI-GO concentrations before aging (dash lines represent the fitted lines using the Herschel–Bulkley model).

Rheological modeling is crucial for characterizing the flow behavior of drilling fluids, which, in turn, is essential for optimum wellbore hydraulics management.^59^Fig. 6 presents the rheogram, depicting the shear stress-shear rate relationship of the drilling fluids with varying PEI-GO concentrations before aging. At low shear rates, the drilling fluid's rheological behavior appears unaffected by PEI-GO concentrations. However, at high shear rates, increasing PEI-GO concentrations markedly influence the drilling fluid's rheological behavior, evident from the differences in shear stress among the concentrations. Table 3 compiles parameters derived from fitting various rheological models, including the Bingham plastic, power law, and Herschel–Bulkley models, all based on the shear stress-shear rate relationship. In this study, the Herschel–Bulkley model stands out as the most suitable choice for describing the shear stress-shear rate curve, as indicated by higher *R*^2^ values approaching 1 and considerably lower root-mean-square error (RMSE) values nearing 0. It surpasses both the power-law and Bingham plastic models in terms of fitting accuracy. This accentuates the Herschel–Bulkley model's superior accuracy in portraying the rheological behavior of the drilling fluids investigated in this study.

**Table 3:** Calculated parameters of Bingham plastic, power law, and Herschel–Bulkley models for base drilling fluid with different PEI-GO concentrations

Rheological models	Parameters	Base	0.1 wt% PEI-GO	0.2 wt% PEI-GO	0.3 wt% PEI-GO	0.4 wt% PEI-GO	0.5 wt% PEI-GO
(a) Bingham-plastic	*τ*_0_ (Pa)	5.5883	6.3535	6.6181	7.3694	7.9713	8.2028
	*τ*_p_ (Pa)	0.0123	0.0132	0.0154	0.0166	0.0222	0.0249
	*R* ^2^	0.8985	0.8959	0.9022	0.8966	0.9163	0.9390
	RMSE	1.7862	1.9391	2.1849	2.4236	2.8964	2.7347
(b) Power-law	*K*	2.1290	2.5325	2.4104	2.7631	2.4998	2.3536
	*n*	0.2936	0.2810	0.3056	0.2979	0.3465	0.3686
	*R* ^2^	0.9923	0.9914	0.9902	0.9934	0.9920	0.9855
	RMSE	0.4919	0.5562	0.6903	0.6133	0.8958	1.3342
(c) Herschel–Bulkley	*τ*_0_ (Pa)	2.4096	2.8814	2.9224	3.0214	3.2683	4.2512
	*K*	0.7695	0.8534	0.8394	1.0681	0.9657	0.6376
	*n*	0.4224	0.4178	0.4403	0.4181	0.4702	0.5416
	*R* ^2^	0.9996	0.9997	0.9985	0.9998	0.9989	0.9992
	RMSE	0.1293	0.1173	0.3155	0.1166	0.3794	0.3641

In Table 3, it's evident that as the PEI-GO concentration increased, both the fluid behavior index (*n*) and fluid consistency coefficient (*K*) exhibited fluctuations, with distinct highest and lowest values. The arrangement of *n* values, from lowest to highest, was 0.1 < 0.3 < base < 0.2 < 0.4 < 0.5 wt%, while for *K* values, it was arranged from highest to lowest as 0.3 > 0.4 > 0.1 > 0.2 > base> 0.5 wt%. A lower *n* value indicated a more pronounced non-Newtonian characteristic in the drilling fluid, which is desirable for achieving effective shear-thinning behavior and enhanced cutting transport, with a corresponding increase in the *K* value enhanced cutting transport.^58^ Therefore, it can be concluded that 0.3 wt% PEI-GO in base drilling fluid consistently performed well in terms of rheological behavior, enhancing the shear-thinning behavior and overall rheological properties. In contrast, the highest PEI-GO concentration, 0.5 wt%, was less favorable in terms of rheological behavior, likely due to the adverse effects of excessive PEI-GO concentration, possibly leading to agglomeration and reduced drilling fluid performance.

The results indicate that the 0.3 wt% PEI-GO consistently displayed significantly higher *K* values compared to the base drilling fluid across all temperature conditions. The higher values of *K* indicate greater viscosity, which promotes efficient transport of cuttings from the borehole to the surface; conversely, a lower *K* may result in cuttings settling owing to gravity.^60^ Furthermore, for samples before aging, the *n* values of PEI-GO were slightly lower than the base drilling fluid. However, as the temperature changes to 80 and 160 °C, the difference between the base drilling fluid and the 0.3 wt% PEI-GO becomes significantly pronounced. It can be observed that 0.3 wt% PEI-GO maintained a low *n* value under both low and high temperature conditions. A lower *n* value improves the drilling fluid's ability to transport cuttings effectively, emphasizing the importance of a lower *n* value for better cuttings carrying capacity.^60^ Overall, drilling fluids with higher *K* values and lower *n* values have a better ability to suspend solid particles and cleaning borehole.^61^ Collectively, these findings highlight the ability of the base drilling fluid with 0.3 wt% PEI-GO to retain and suspend solid particles, even at high temperature conditions.

**Fig. 7:**
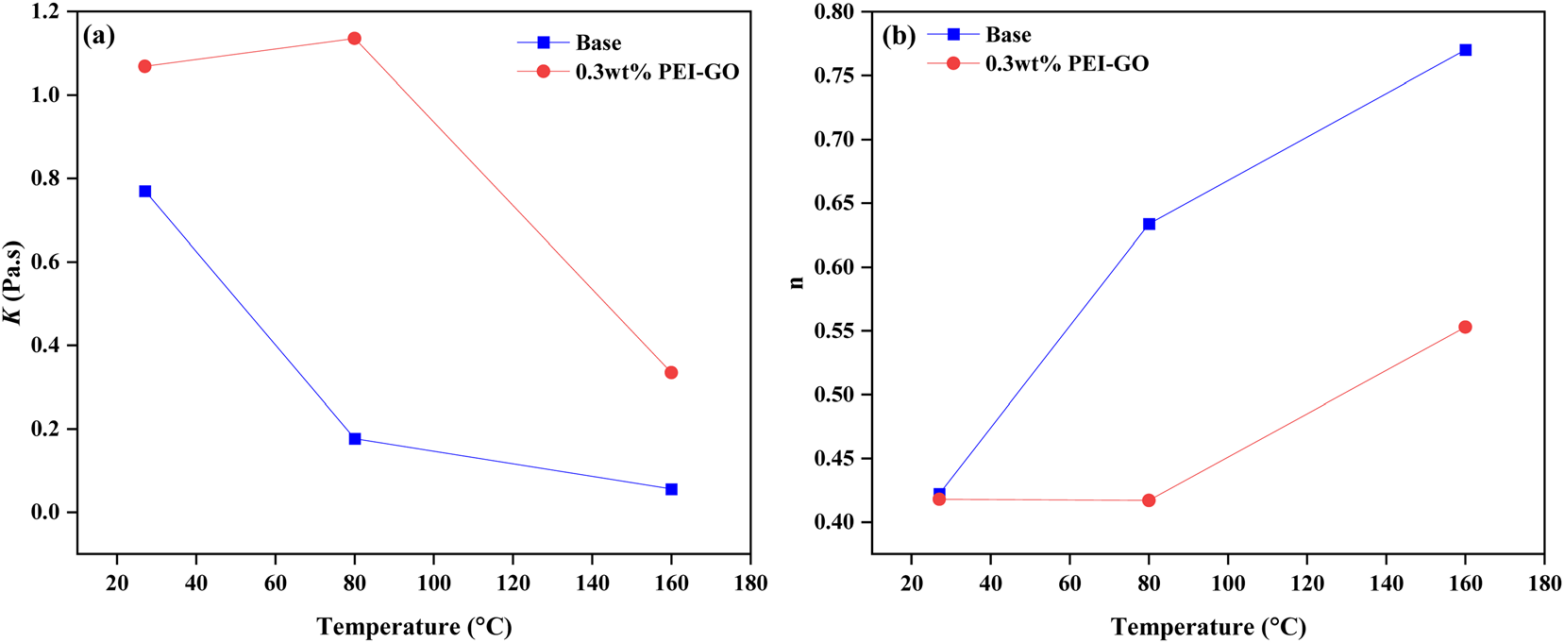
Plot of base drilling fluid and base drilling fluid with 0.3 wt% PEI-GO at different temperatures for (a) fluid consistency coefficient (*K*) and (b) fluid behavior index (*n*).

The influence of temperature on the rheological characteristics of the drilling fluid was examined by analyzing the Herschel–Bulkley model parameters, *K* and *n* values. These parameters are intrinsically linked, with alterations in one directly impacting the other.^62^ The investigation encompassed both the base drilling fluid and the variant containing 0.3 wt% PEI-GO, as depicted in Fig. 7 and Table 4. The outcomes reveal a discernible pattern, as temperature ascends, *K* values decline while *n* values ascend, a phenomenon observed in both the base drilling fluid and the 0.3 wt% PEI-GO. This trend signifies a reduced tendency of the drilling fluid to suspend solid particles at elevated temperatures.^63^

**Table 4:** Calculated parameters for base drilling fluid and base drilling fluid added with 0.3 wt% PEI-GO under varying temperatures *via* the Herschel–Bulkley model

Samples	Parameters	Before aging	After aging-80 °C	After aging-160 °C
Base drilling fluid	*τ*_0_ (Pa)	2.4096	2.5546	2.8359
	*K*	0.7695	0.1769	0.0559
	*n*	0.4224	0.6336	0.7701
	*R* ^2^	0.9996	0.9999	0.9922
	RMSE	0.1293	0.0198	0.4960
0.3 wt% PEI-GO	*τ*_0_ (Pa)	3.0214	1.7776	2.9236
	*K*	1.0681	1.1348	0.3344
	*n*	0.4181	0.4171	0.5530
	*R* ^2^	0.9998	0.9974	0.9994
	RMSE	0.1166	0.4662	0.1733

### 3.4 Filtration properties

**Fig. 8:**
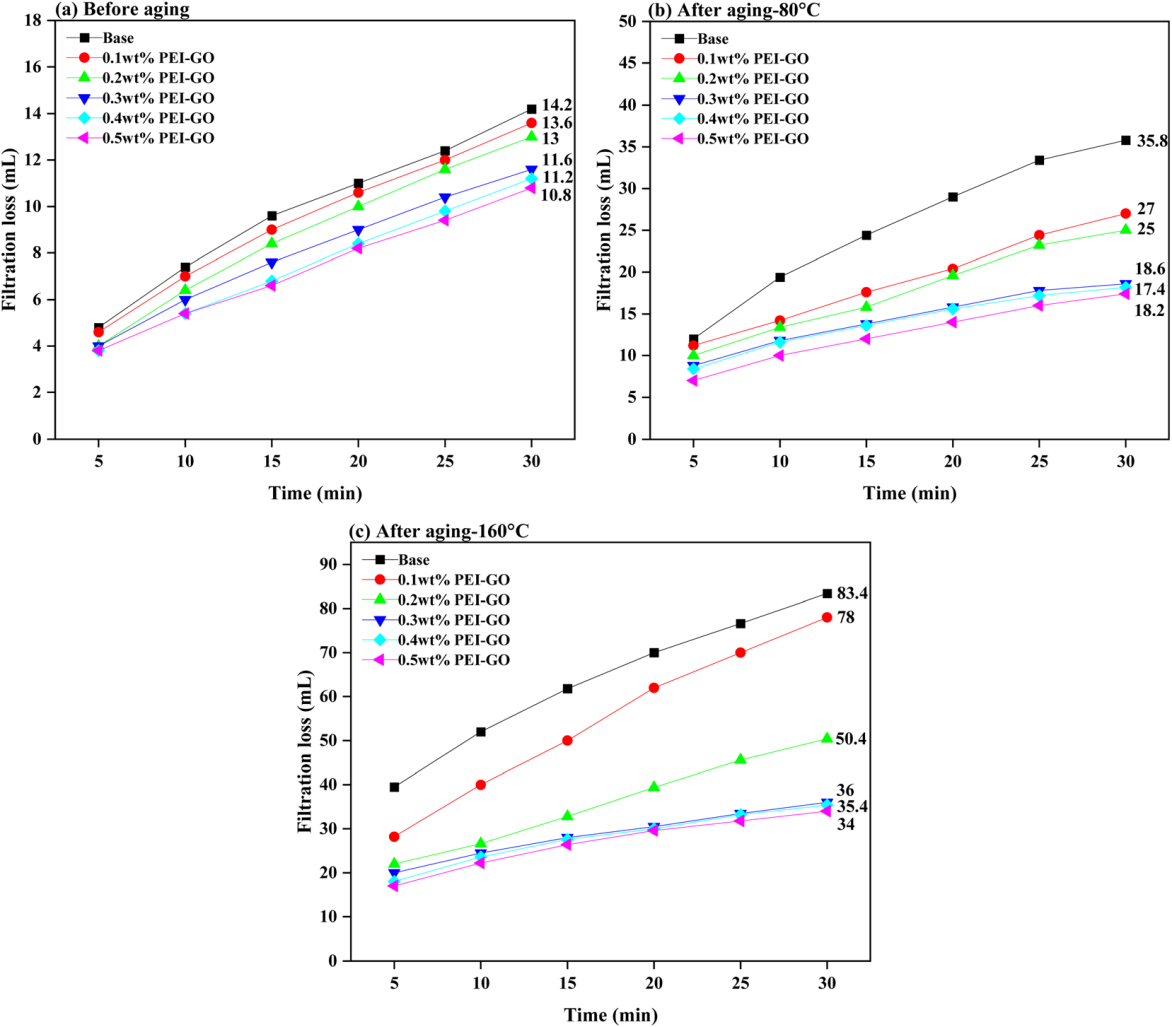
API filtration volume comparison between the base drilling fluid and the base drilling fluid with varying concentrations of PEI-GO under varying temperatures: (a) before aging, (b) after aging at 80 °C, and (c) after aging at 160 °C.

Fig. 8 illustrates the influence of PEI-GO on the filtration properties of the base drilling fluid. The experimental results indicated that the incorporation of PEI-GO resulted in a significant decrease in the amount of API filtration loss seen in the base drilling fluid. Furthermore, it is noteworthy that an increase in the ageing temperature resulted in a more noticeable disparity in the API filtration volume between the base drilling fluid and the base drilling fluid added with PEI-GO. For instance, the percentage difference in API filtration volume for the 0.3 wt% PEI-GO compared to base drilling fluid was only 18.31% before aging. However, after aging at 80 and 160 °C, this difference increased to 48.04% and 56.83%, respectively. These results showed that PEI-GO reduces fluid loss better than base drilling fluid after aging at temperature up to 160 °C. PEI-GO enhanced base drilling fluid by maintaining stable rheology and reducing filtrate loss volume at high temperatures, making it a better additive option for high-temperature applications.

**Fig. 9:**
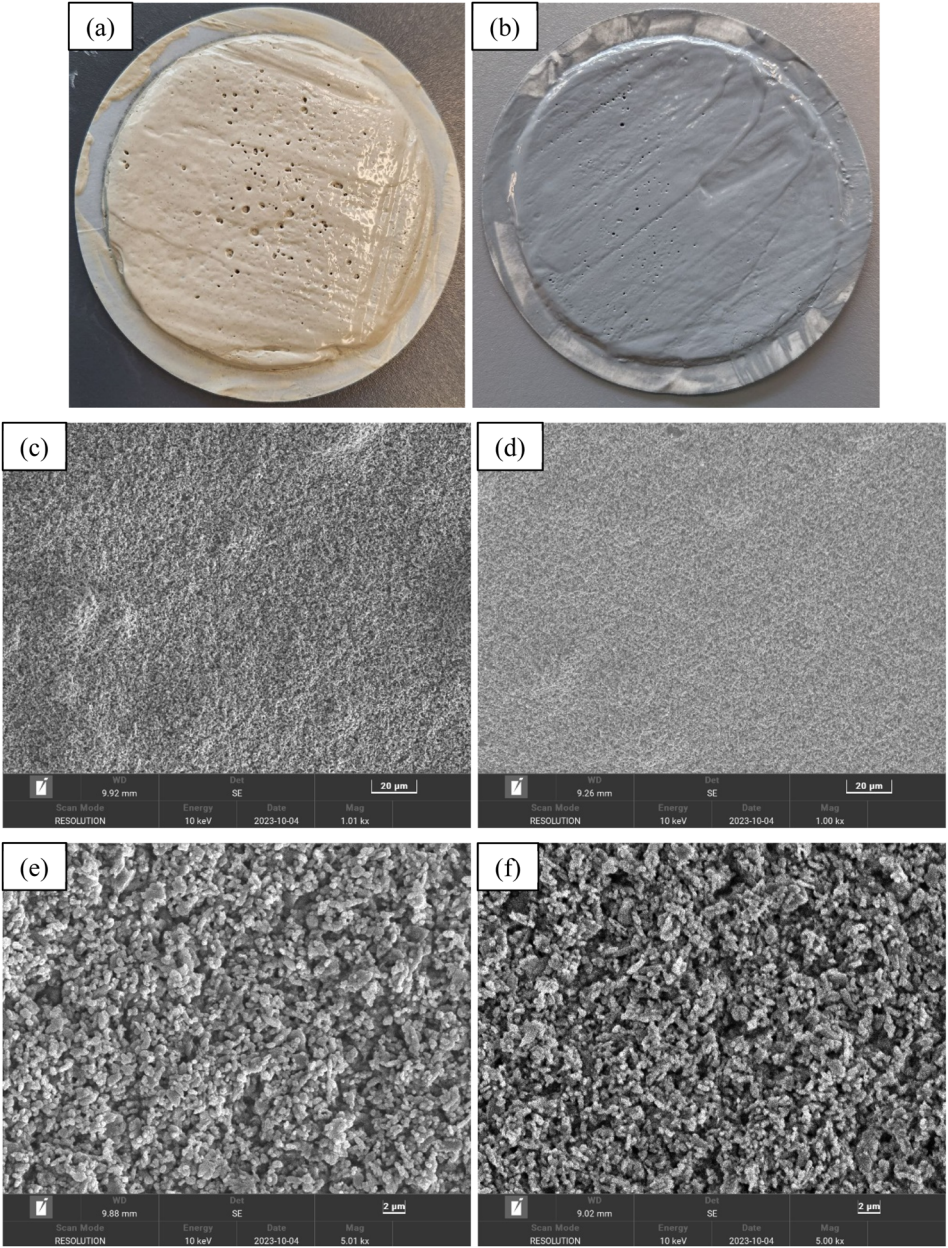
Images of filter cakes and SEM analysis formed by the base drilling fluid (a, c and e) and base drilling fluid containing 0.3 wt% PEI-GO (b, d and f) after aging at 160 °C.

Fig. 9 presents visual and scanning electron microscopy (SEM) images of filter cakes formed from the base drilling fluid and base drilling fluid containing 0.3 wt% PEI-GO after aging at 160 °C. The visual of these filter cakes, as shown in Fig. 9a and b, indicates that the base drilling fluid exhibits larger pores on the surface of the filter cake in comparison to the 0.3 wt% PEI-GO-containing drilling fluid, which displays fewer and smaller pores on its filter cake surface. Furthermore, the filter cake created by base drilling fluid is significantly thicker compared to that of the 0.3 wt% PEI-GO-containing fluid. To be specific, the thickness of the base drilling fluid filter cake measures 4.32 mm, whereas the PEI-GO filter cake is notably thinner at 1.24 mm. It is noteworthy that an excessively thick filter cake can lead to wellbore-related issues, including tight holes, causing differential sticking, and inducing elevated pressure surges.^3^ A closer examination through SEM at 1000- and 5000 times magnification reveals the distinct characteristics of each filter cake, as shown in Fig. 9c–f. The filter cake from the drilling fluid containing 0.3 wt% PEI-GO displays a flatter and smoother surface in comparison to the filter cakes from the base drilling fluid. This is attributed to the presence of the PEI-GO nanocomposite, which forms a hydrophobic film that seals the pores, preventing fluid flow and resulting in a thinner and densely packed filter cake with reduced filtration loss volume compared to the base drilling fluid.^16^

The impact of PEI-GO concentration on filtration loss reduction is also clearly demonstrated in Fig. 8. The addition of 0.1 and 0.2 wt% of PEI-GO led to a decrease in filtration loss of 4.23% and 8.45%, respectively, compared to the base drilling fluids in the pre-aging sample. This reduction effect became more pronounced when 0.3 to 0.5 wt% PEI-GO were employed, resulting in filtration loss reductions between 18.31 to 23.94%. This trend remained consistent when the samples were subjected to elevated temperatures of 80 and 160 °C, with 0.3–0.5 wt% PEI-GO demonstrating the highest percentages of fluid loss reduction which ranging between 48.04–51.40% and 60.43–61.96%. This finding indicates that a concentration range of 0.3 to 0.5 wt% PEI-GO had high thermal resistance.

**Fig. 10:**
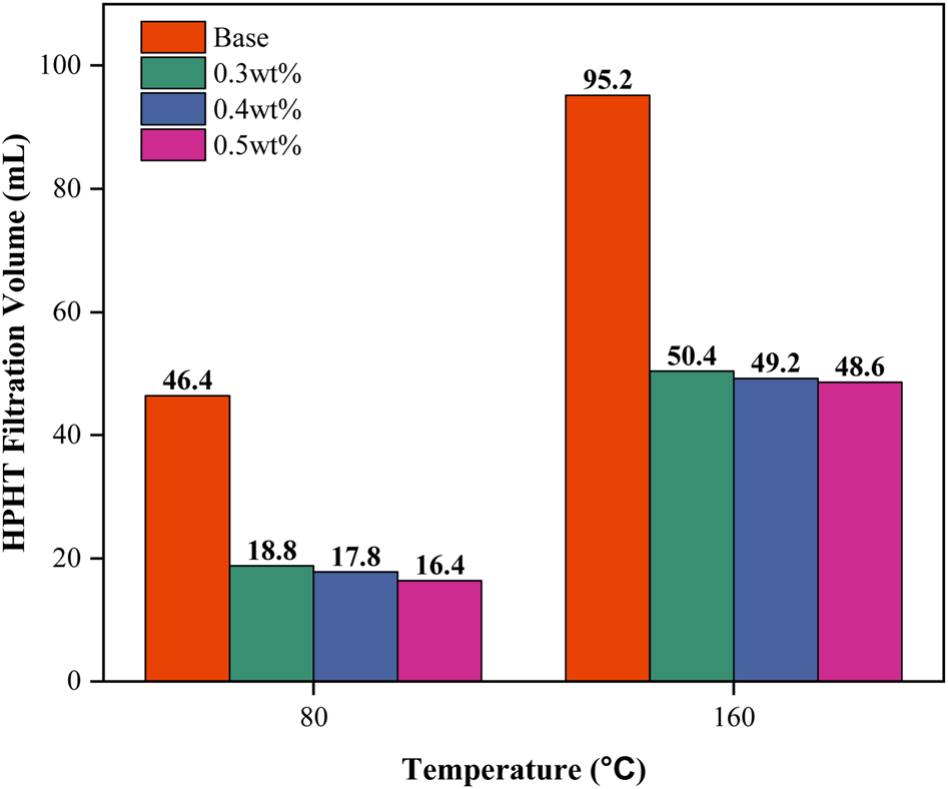
Filtration volumes under high-pressure, high-temperature (HPHT) conditions for the base drilling fluid from 0.3 to 0.5 wt% PEI-GO at various temperatures.

To further validate the temperature resistance between 0.3 to 0.5 wt% PEI-GO, high-pressure high-temperature (HPHT) filtration tests were conducted at a differential pressure of 500 psi and temperatures of 80 and 160 °C. As illustrated in Fig. 10, the HPHT filtration volume of 0.3 and 0.5 wt% PEI-GO consistently remained lower than that of base drilling fluid. In contrast to base drilling fluid, as temperatures increased, 0.3 to 0.5 wt% PEI-GO exhibited substantially lower filtration volumes, indicating their capability to withstand temperatures as high as 160 °C. This is supported by the high thermal stability characteristics of PEI-GO nanocomposite as identified in the TGA analysis. Moreover, when comparing the API and HPHT filtration loss between 0.3–0.5 wt% PEI-GO, the difference between them was minimal, less than 5%. This finding indicates that the augmentation of PEI-GO concentration did not provide a substantial improvement in the ability to reduce fluid loss. In some cases, a further increase in nanoparticle concentration adversely impacted rheological properties and filtration loss performance.^47,60^ This was attributed to nanoparticle agglomeration, mirroring the behavior of larger particles and resulting in an elevated level of filtration loss.^64^

The rheological characteristics and filtration performance of a drilling fluid containing 0.3 wt% PEI-GO align with the operational requirements of drilling fluids. Thus, taking into account both cost considerations and performance, this study concludes that the most optimal concentration is 0.3 wt% PEI-GO. This finding is in line with the enhancement of rheological and filtration loss properties observed at optimal nanoparticle concentrations, as reported by Arain *et al.*^47^

### 3.5 Influence of high salinity condition on PEI-GO performance in drilling fluid

The introduction of sodium (Na^+^) and calcium (Ca^2+^) ions has been seen to disturb the diffusion double-layer arrangement of bentonite, resulting in a fast decline in the rheological and filtration characteristics of the solution composed of bentonite.^65^ This flocculation phenomenon might result in a thick filter cake, which often leads to differential pressure sticking in permeable zones. Table 5 summarizes the rheological properties (PV, YP, and YP/PV) of the 0.3 wt% PEI-GO drilling fluid before and after aging under elevated concentrations of NaCl and CaCl_2_.

**Table 5:** Rheological values of the base drilling fluid containing 0.3 wt% PEI-GO for different concentrations of NaCl and CaCl_2_ before aging and after aging at 160 °C

Salt concentration (wt%)	Before aging			After aging – 160 °C		
	PV (mPa s)	YP (Pa)	YP/PV (1/s)	PV (mPa s)	YP (Pa)	YP/PV (1/s)
0 wt% NaCl	10.0	12.0	1.20	9.5	8.5	0.89
5 wt% NaCl	10.5	12.25	1.17	8.5	6.0	0.71
15 wt% NaCl	12.0	10.0	0.83	5.0	3.5	0.70
20 wt% NaCl	12.0	9.75	0.81	4.5	3.5	0.78
25 wt% NaCl	13.0	5.5	0.42	6.0	1.5	0.25
0 wt% CaCl_2_	10.0	12.0	1.20	9.5	8.5	0.89
5 wt% CaCl_2_	8.5	9.5	1.12	5.0	3.5	0.70
10 wt% CaCl_2_	9.0	9.75	1.08	5.0	3.0	0.60
15 wt% CaCl_2_	14.5	3.0	0.21	6.5	1.25	0.19

In general, the augmentation of NaCl and CaCl_2_ concentrations resulted in a reduction in the rheological characteristics of the drilling fluid. However, the impact on PV and YP was minimal with increasing salt concentrations. It was evident that the YP/PV values significantly decreased at salt concentrations of 25 wt% NaCl and 15 wt% CaCl_2_, indicating the detrimental effect of higher salt concentrations on the shear-thinning behavior and carrying capacity of drilling fluids. After high-temperature aging, the rheological properties of the drilling fluids decreased at the same NaCl and CaCl_2_ concentrations, indicating that aging temperature had a more substantial influence on PEI-GO compared to salt concentrations. Nevertheless, the YP/PV value of the PEI-GO drilling fluid remained stable before and after aging at 160 °C, indicating that it was able to maintain the fluid's cuttings carrying capacity and wellbore cleaning efficiency even under high salinity conditions.^66^ It is worth noting that, when comparing the two salt types, CaCl_2_ had a more pronounced adverse effect on the performance of the PEI-GO drilling fluid compared to NaCl.

**Fig. 11:**
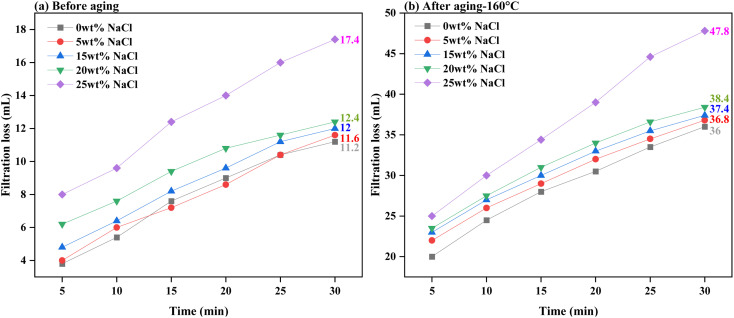
Filtration loss of the base drilling fluid added with 0.3 wt% PEI-GO under different NaCl concentrations: (a) before aging and (b) after aging at 160 °C.

**Fig. 12:**
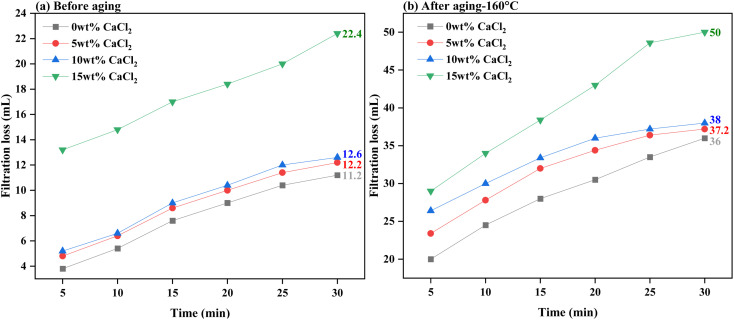
Filtration loss of the base drilling fluid added with 0.3 wt% PEI-GO under different CaCl_2_ concentrations: (a) before aging and (b) after aging at 160 °C.

Fig. 11 and 12 present the evaluation of how well base drilling fluid enriched with 0.3 wt% PEI-GO, withstands high salinity environments of NaCl and CaCl_2_ in terms of filtration loss volume. This assessment is conducted both before and after subjecting the fluids to aging at 160 °C. Fig. 11a demonstrates that the addition of 0.3 wt% PEI-GO significantly reduces filtration loss volume, even when tested for NaCl contamination at concentrations up to 20 wt%, prior to aging. This observation remains consistent after aging the drilling fluid sample at 160 °C, as depicted in Fig. 11b. However, at a NaCl concentration of 25 wt%, both filtration loss volume and rate sharply increase for both temperature conditions. Turning to Fig. 12a and b, a slight increase in filtration loss volume is noticeable at 5 and 10 wt% CaCl_2_, but it substantially rises at 15 wt% CaCl_2_. It can be concluded that 0.3 wt% PEI-GO exhibits excellent tolerance to NaCl and CaCl_2_, particularly at concentrations of 20 wt% and 10 wt%, respectively. This makes it a suitable choice for application in high-salinity formations, even at high temperatures up to 160 °C. The outcomes of this investigation regarding the impact of NaCl and CaCl_2_ salinity on rheological and filtration loss properties align with findings reported in the literature.^66^

## 4. Conclusion

The polymerization of low molecular weight PEI with GO was successfully performed, demonstrating the formation of PEI-GO nanocomposite, as confirmed by FTIR analysis. The high thermal resistance of the nanocomposite was evident through TGA analysis. This study comprehensively investigated the rheological properties, rheological modeling, filtration properties, and the influence of high-temperature and high-salinity conditions on drilling fluid performance enhanced with varying concentrations of PEI-GO nanocomposite. The experimental findings lead to the following conclusions:

• The rheological properties evaluated in this study: PV, YP and YP/PV were improved with the increasing concentration up to 0.5 wt% of PEI-GO in the water-based drilling fluids.

• The Herschel–Bulkley model emerged as the most accurate in describing drilling fluid rheological behavior, as evidenced by the best fit to the shear stress-shear rate curve. Additionally, the fluid behavior index (*n*) and fluid consistency coefficient (*K*) demonstrated improvements when using base drilling fluid containing PEI-GO, indicating superior shear-thinning behavior and overall rheological properties.

• PEI-GO effectively reduced both API and HPHT filtration loss volumes of the base drilling fluid. Visual and SEM analyses revealed that PEI-GO led to the formation of a thinner and denser filter cake, further enhancing filtration loss reduction.

• This study determines that a concentration of 0.3 wt% of PEI-GO is the most effective, indicating that a modest quantity of the nanocomposite is sufficient to enhance the rheological and filtration loss properties of water-based drilling fluids.

• Furthermore, PEI-GO exhibited excellent resistance to NaCl and CaCl_2_, particularly up to concentrations of 20 wt% and 10 wt%, respectively. The PEI-GO's outstanding rheology and filtration properties are due to its electrostatic interaction with clay particles through hydrogen and ionic bonding, resulting in pore plugging within the filter cake. This, in turn, prevented water infiltration and reduced filtration loss volume, thereby mitigating wellbore instability issues.

• The high thermal stability of PEI-GO is evident according to the assessment of PEI-GO performance in rheological and filtration loss properties when compared to the base drilling fluids, even at temperatures as high as 160 °C.

In this study, PEI-GO nanocomposite consistently maintained stable filtration loss reduction and rheological properties, even under high-salinity conditions and elevated temperatures, making it a suitable choice for challenging drilling environments. An extension of this research work could explore the applicability of PEI-GO as a shale inhibitor agent under harsh wellbore conditions, further enhancing its versatility in drilling operations.

## Author contributions

Abdul Hazim Abdullah: conceptualization, data curation, formal analysis, investigation, methodology, visualization, writing – original draft. Syahrir Ridha: funding acquisition, project administration, resources, software, supervision, validation, writing – review & editing. Dzeti Farhah Mohshim: formal analysis, methodology, writing – review & editing. Mohd Azuwan Maoinser: funding acquisition, formal analysis, methodology, writing – review & editing.

## Conflicts of interest

The authors declare that they have no known competing financial interests or personal relationships that could have appeared to influence the work reported in this paper.

## Supplementary Material
